# New ECCO model documents for Material Deposit and Transfer Agreements in compliance with the Nagoya Protocol

**DOI:** 10.1093/femsle/fnaa044

**Published:** 2020-03-09

**Authors:** Gerard Verkley, Giancarlo Perrone, Mery Piña, Amber Hartman Scholz, Jörg Overmann, Aurora Zuzuarregui, Iolanda Perugini, Benedetta Turchetti, Marijke Hendrickx, Glyn Stacey, Samantha Law, Julie Russell, David Smith, Nelson Lima

**Affiliations:** 1 Westerdijk Fungal Biodiversity Institute, Uppsalalaan 8, 3584 CT Utrecht, The Netherlands; 2 Institute of Sciences of Food Production (ISPA), National Research Council (CNR), Via Amendola 122/O, 70126 Bari, Italy; 3 CRBIP-Biological Resource Centre, Department of Microbiology, Institut Pasteur, 25-28 Rue du Docteur Roux, 75015 Paris, France; 4 German Collection of Microorganisms and Cell Cultures (DSMZ), Inhoffenstrasse 7B, 38124 Braunschweig, Germany; 5 Spanish Type Culture Collection (CECT), Edificio 3 CUE, Parc Científic Universitat de València, Catedrático Agustín Escardino 9, 46980 Paterna (Valencia), Spain; 6 Mycotheca Universitatis Taurinensis (MUT), Department of Life Sciences and Systems Biology, University of Torino, Viale P.A. Mattioli 25, 10125 Torino, Italy; 7 Industrial Yeasts Collection (DBVPG), Department of Agriculture, Food and Environmental Sciences, University of Perugia, Borgo XX Giugno 74, I-06121 Perugia, Italy; 8 BCCM/IHEM Fungal Collection, Mycology & Aerobiology, Sciensano, Juliette Wytsmanstraat 14, B-1050 Brussels, Belgium; 9 International Stem Cell Banking initiative, Barley, Hertfordshire, SG88HZ, UK; 10 National Collection of Industrial, Food and Marine Bacteria (NCIMB), Ferguson Building, Craibstone Estate, Bucksburn AR21 9YA, Aberdeen, UK; 11 Public Health England (PHE) Culture Collections, Porton Down, SP4 0JG Salisbury, UK; 12 CABI, Bakeham Lane, TW20 9TY Egham, UK; 13 Micoteca da Universidade do Minho (MUM), CEB-Centre of Biological Engineering, University of Minho, Campus de Gualtar, 4710-057 Braga, Portugal

**Keywords:** EU regulation No. 511/2014, access and benefit sharing (ABS), European culture collections’ organisation (ECCO), convention on biological diversity (CBD), material deposit agreement (MDA), material transfer agreement (MTA)

## Abstract

The European Culture Collections’ Organisation presents two new model documents for Material Deposit Agreement (MDA) and Material Transfer Agreement (MTA) designed to enable microbial culture collection leaders to draft appropriate agreement documents for, respectively, deposit and supply of materials from a public collection. These tools provide guidance to collections seeking to draft an MDA and MTA, and are available in open access to be used, modified, and shared. The MDA model consists of a set of core fields typically included in a ‘deposit form’ to collect relevant information to facilitate assessment of the status of the material under access and benefit sharing (ABS) legislation. It also includes a set of exemplary clauses to be included in ‘terms and conditions of use’ for culture collection management and third parties. The MTA model addresses key issues including intellectual property rights, quality, safety, security and traceability. Reference is made to other important tools such as best practices and code of conduct related to ABS issues. Besides public collections, the MDA and MTA model documents can also be useful for individual researchers and microbial laboratories that collect or receive microbial cultures, keep a working collection, and wish to share their material with others.

## Teaser

To facilitate access to biological materials in compliance with legal requirements, including the Convention on Biological Diversity and the Nagoya Protocol, the European Culture Collections’ Organisation (ECCO) developed two new model documents for Material Deposit Agreement (MDA) and Material Transfer Agreement (MTA).

## INTRODUCTION

Culture collections provide important services to science and society by preserving and supplying biological materials and associated data for research, education and industrial applications, and by offering expert knowledge and technology for the characterization and handling of the biological materials. They perform these services under increasing regulatory pressures related to the management of microorganisms i.e. biosecurity, food safety and access and benefit-sharing (ABS) under the Nagoya Protocol (NP 2011). The Convention on Biological Diversity (CBD 1992) entered into force on 29th December 1993. Aims of this treaty were to conserve biological diversity, and to foster its sustainable use and the fair and equitable sharing of benefits arising from its utilization. The basic principle is that the CBD recognizes the sovereign rights of countries over their own ‘genetic resources’. According to the CBD genetic resources are defined as ’genetic material of actual or potential value’ and genetic material is defined as ‘any material of plant, animal, microbial or other origin containing functional units of heredity’. These can include, for example, living and dead biological specimens (e.g. fungarium specimens), DNA extracts, and other derivatives (e.g. secondary metabolites). Thus, countries that have become party to the treaty can grant access to their genetic resources (and associated traditional knowledge) and determine the conditions for access. Human genetic resources (human tissue, blood) are not covered by the CBD (1992) and Nagoya Protocol (NP 2011).

The European Culture Collections’ Organisation (ECCO, www.eccosite.org) was founded as a non-profit organization in 1981 with the mission to support the interests of European culture collections of microorganisms (bacteria, archaea, fungi, viruses and cell lines of plant, animal and human origin) and help them to improve scientific and technical standards in close collaboration with the World Federation for Culture Collections (WFCC, www.wfcc.info). Furthermore, it endeavours to inform and support users of material maintained by microbial collections. In order for culture collections to qualify for corporate membership in ECCO, they: (i) have to be registered in the WFCC-MIRCEN World Data Centre for Microorganisms database (WDCM, www.wdcm.org), (ii) have an online searchable strain catalogue of their holdings, and (iii) supply their material to the scientific user community. The number of collections that became member of ECCO has steadily grown over the years and at present there are 76 corporate members from 26 European countries. The total number of strains preserved by all ECCO collections is estimated at 500 000. ECCO meetings are held annually and hosted by one of the member collections. ECCO is often invited by the organisers of congresses in the microbial domain to (co-)organise special symposia and round tables, for example during the International Conference on Culture Collections (ICCC) of the WFCC and Federation of European Microbiological Societies (FEMS) congresses of European microbiologists and make presentations in other European regional meetings and beyond. In addition, ECCO is an important stakeholder in other initiatives, for example its participation on the first Food and Agriculture Organization (FAO) report on The State of the World's Biodiversity for Food and Agriculture (FAO 2019).

Here, we present new model documents for Material Deposit and Transfer Agreements with the aim to support legal clarity regarding Nagoya Protocol issues on the cultures supplied by collections. We believe that agreements based on these model documents will facilitate effective and efficient exchange of materials within Europe and with the rest of the world.

## THE FIRST ECCO ‘CORE’ MTA

Since the CBD came into force, scientific organizations and collections preserving genetic resources *ex-situ* have expressed their concerns to legislators and policy makers about the negative consequences of implementing too rigid and restrictive access and benefit sharing regimes (Overmann and Scholz 2017). Both public collections as well as science in general depend on availability and exchange of research materials and information (McCluskey *et al*. 2017; Smith *et al*. 2017). An operational framework for *ex-situ* collections involving the use of a material transfer agreement (MTA) would help to overcome the legal uncertainty the providing collections and the recipients of materials are facing. Therefore, in 2005 ECCO established a working group to define and describe the commonly agreed ‘core’ content (i.e. content considered to be essential) of an MTA to be used for the supply of samples by ECCO collections. Traceability and fair and equitable sharing of benefits were addressed in this Core MTA, together with other key items for public collections and users of the materials supplied, viz. intellectual property rights, quality, safety and security (Janssens *et al*. 2009). ECCO collections were free to extend the Core MTA as appropriate or necessary under their own legal framework, whilst the supply by the various ECCO collections would remain under the same essential conditions. The ECCO Core MTA was endorsed by the ECCO annual general meeting in 2009. Since then, it has been incorporated by many microbial collections and other organizations dealing with transfer of material and even used by collections outside Europe.

## THE NAGOYA PROTOCOL AND ACCESS TO GENETIC RESOURCES

The ‘Nagoya Protocol on access to genetic resources and the fair and equitable sharing of benefits arising from their utilization’ (NP 2011) was agreed upon by the Parties to the CBD in 2010. The Protocol entered into force on October 12th, 2014, and thus far 120 countries (as of November 2019) have become a Party. Parties are free to determine whether access to their genetic resources will be subject to such requirements or not, or only for specific genetic resources or areas, issuing their own national regulations. If a Nagoya Party decides to regulate access to its genetic resources, it may require (i) formal permission from the competent authority in that country, the ‘Prior Informed Consent’ (PIC) and (ii) settling between provider and user the terms for use, the ‘Mutually Agreed Terms’ (MAT). Thus access can be entirely ‘free’ ’, i.e. not regulated under appropriate legislation (no PIC needed) in the context of ABS, as, for instance, is the case in the Netherlands, Germany and UK, although permits to collect materials may still be required under environmental protection laws, from landowners, or national parks, etc. In Portugal access is not regulated in the homeland but, in contrast, in Madeira and Azores islands requires an access permit. In practice, a permit to collect and export, and the terms agreed as regards the use of the genetic resource may be either in separate documents (PIC, MAT or in an online standardized form called the internationally recognized certificate of compliance (IRCC)) as described above or they may be integrated into a single comprehensive agreement (often called a material transfer agreement (MTA)).

A considerable number of countries have not yet completed the process of designing and implementing national ABS legislation. In accordance with Article 14(2) of the Nagoya Protocol, Parties are expected to provide information about their ABS legislation on the ABS Clearing-House database (ABSCH 2019), and information on access permits issued by competent authorities in the form of internationally recognized certificates of compliance (IRCC) records. Unfortunately, it appears that not all countries will publish and use IRCCs but instead rely on highly individualized documentation. Although the situation has improved since 2014, many countries have yet to upload most crucial information onto this ABSCH. The European Union was relatively early with designing and implementing the Regulation (EU) No 511/2014 (EU 2014) (henceforth referred to as ‘the EU Regulation’), which entered into force on October 12th, 2014. The EU Regulation governs user compliance measures and benefit sharing within the Union. It does not deal with either access to the genetic resources of the Member State, the mechanism of monitoring compliance, or legal consequence of infringement in the Member States. The EU Regulation is complemented by the Commission Implementing Regulation (EU) 2015/1866 (EU 2015) providing detailed rules as regards the register of collections, monitoring user compliance and best practices. Also important for users of genetic resources in Europe, although not legally binding, is the European Commission Guidance document on the scope of application and core obligations (EC 2016). An update as well as more detailed sectoral guidance in some form are expected to become available later. Some Member States have been slow to define their compliance officers and authorities at the national level, but it should be remembered that the EU Regulation is already in force in all Member States.

## WHY AN MDA AND WHY A NEW MTA?

Nagoya Protocol articles 19 and 20 encourage the development of tools intended to support and tailor implementation to specific stakeholder groups, namely model contractual clauses, voluntary codes of conduct, guidelines, best practices and standards. In response to these articles, the Microbial Resources Research Infrastructure (MIRRI, http://www.mirri.org/) of which several ECCO members are partner, developed a ‘Best Practice Manual on Access and Benefit Sharing’ (Verkley *et al*. 2016) to help collections preserving microbial genetic resources *ex-situ* to reach compliance. This tool was listed on the ABS-Clearing House information resource ‘Model Contractual Clauses, Codes of Conduct, Guidelines, Best Practices and/or Standard’. Other notable tools listed there include TRUST (also for microorganisms) and the Consortium of European Taxonomic Facilities ‘Code of Conduct and Best Practice for Access and Benefit Sharing and Material Transfer Agreements’ for non-microbial collections (CETAF 2019).

In 2018, the CBD Secretariat requested that ECCO upload the ECCO Core MTA on the ABSCH, because various stakeholders had mentioned that they use this MTA or considered it relevant in response of the Secretariat's call ‘Notification 2017-104’. By then, ECCO experts had already started to create a new model MTA for supply, as the ECCO Core MTA of 2009 was evidently in need of an update. ECCO also decided to design a model for deposit of material in the public collection (MDA) because such a document would help collections to make sure that relevant ABS information would be obtained from the depositors, so that it could be checked at the right time, when the new material is offered for deposit in the public collection. Drafts of the MTA and MDA model documents were discussed during the ECCO Meeting held in Turin, Italy from June 12–14, 2019, and subsequently approved by the ECCO Annual General Meeting.

## WHO CAN USE THEM?

Any public microbial culture collections regardless the size of its holdings can benefit from the ECCO model MDA and MTA, but also many other keepers and users of microbial genetic resources that need to develop a legal operational framework may find these models useful. Scientists in public and private sector laboratories and research departments often accept and keep microbial culture collections, and will also need to comply with relevant ABS requirements if they use these resources in research and/or development in the sense of the Nagoya Protocol, or in case they want to share the materials with other partners while enabling legal clarity for both provider and recipient of the material. The MDA and MTA models can help all these users to compile appropriate legal documents for transfer and to reach compliance.

## HOW TO USE THE MDA AND MTA models

The MDA and MTA model documents are freely available at the ECCO website (www.eccosite.org) and provide guidance to compile an institute's own MDA and MTA and with the publication of this paper they will also be uploaded to the ABS Clearinghouse. Importantly, these models are not ready-for-use templates, but rather they require additional input and consideration from the collection holder. Each collection needs to assess if the clauses in these models will fit their specific deposit and material transfer events and internal processes and, especially, requirements of domestic (national) ABS and other legislation.

The definitions of terms in both models are compatible with each other as well as the definitions in the Nagoya Protocol and EU Regulation.

Many culture collections have a long history and a considerable part of their holdings fall outside the temporal scope of the CBD and Nagoya protocol, or were accepted for deposit under certain conditions of the depositors. Each collection has to operate under a complex legal framework and must assess how it implements MDA and MTA tools, and needs to assure that transfer of use rights by the collection to the recipients of these genetic resources is arranged in such a way that it is compliant to all relevant laws and regulations. In certain cases collections might restrict use for commercial purposes but only if this is dictated by the collections’ local legal framework or funding conditions. It is stressed that nothing in the ECCO models provided here is intended to restrict the sovereign rights of countries of origin of genetic resources in any way, nor to dictate how the collections choose to design their clauses pertaining to use of the genetic resources.

### The MDA model

In order to provide legal certainty to both depositor and collection, it needs to be determined if the material concerned is covered by ABS national legislation or regulatory requirements. For this reason, certain information needs to be provided to the collection by the depositor, and this is normally done by completing a deposit form (often this is also referred to as the ‘accession form’). The MDA model (supplementary material 1) provides in Part I ‘Deposit Form’ a list of fields and associated questions that ECCO considers core and important to obtain the information needed to ‘exercise due diligence’ under the EU Regulation.

For example, a collection could use this model to check and update their existing deposit form. The model may also serve as a starting point for making a new deposit form. It does not include fields important to collect scientific or technical information. Such fields will vary with the type of organisms that a collection is able to preserve.

The deposit procedure should also settle other aspects such as the conditions for using the material by the collection or by third parties (clients of the public collection). These aspects can be dealt with in a separate agreement or in a section for terms and conditions in the deposit form. Part II ‘Definitions and Terms and Conditions for deposit in the public collection’ provides a list of definitions and example clauses addressing the issues that ECCO considers important to provide legal clarity. Collections need to check if their national legislation would demand additional or modified clauses to reach compliance.

Collections can consider combining the deposit form with the elements of a material deposit agreement. Such a combined document should not become too long and laborious to complete. Alternatively, collections can use a deposit form and an MDA as separate documents.

### THE MTA model

This model (supplementary material 2) provides a list of definitions and example clauses that ECCO considers core and important in a material transfer agreement between a collection and a recipient, be it a scientist working in a public body, private company, or another collection (so exchange between collections as is included under ‘Legitimate Exchange’ in this model), for various scenarios of material transfer.

Annex 1 (supplementary material 3) provides additional clauses for commercial use, and Annex 2 (supplementary material 4) ‘Description Form’ serves as an example document for listing key data on the strains to be transferred.

## CONCLUSION

It is essential that a common understanding is reached on best practice for microbial culture collections to comply with the Nagoya Protocol and, first and foremost the EU Regulation and its due diligence requirement. Not only do we need to reduce administrative burden for collections but we want to make it a harmonized practice for all microbiologists depositing strains within European collections and clarify the position for research and industry users of microbial strains. The first ECCO MTA was well received and one decade later an update was needed to catch up with developments and ensure compliance with latest regulations. The MDA and MTA model documents (supplementary material 1 and 2) provide detailed clauses and required procedure for compliance but they are not designed as complete model documents as even within Europe the country requirements differ. Furthermore, some issues regarding the interpretation of elements in the EU Regulation and Nagoya Protocol are still being discussed at the level of the EU and CBD, and may be resolved later. For example, it is currently under debate how to deal with digital sequence information (DSI) and the human microbiome. Although it is unlikely that the outcome of these discussions will significantly affect the content of the MDA and MTA models as presented here, ECCO is committed to staying focused on these topics and if necessary will adopt these tools in future. Flexibility is needed to accommodate both these national differences and differences in individual culture collection institutional requirements. Accordingly, the ECCO members have agreed with the content at its 2019 ECCO annual general meeting and consider the value of the documents to extend globally beyond Europe.

## Supplementary Material

fnaa044_Supplemental_Files

## References

[bib1] ABSCH *Access and Benefit Sharing Clearing House* , 2019. https://absch.cbd.int/ (11 March 2020, date last accessed).

[bib2] CBD *Convention on Biological Diversity*. United Nations, 1992. https://www.cbd.int/doc/legal/cbd-en.pdf (11 March 2020, date last accessed).

[bib3] CETAF- Consortium of European Taxonomic Facilities. *Code of Conduct and Best Practices on Access and Benefit-Sharing and Material Transfer Agreement Templates*, 2019. https://cetaf.org/services/natural-science-collections-and-access-and-benefit-sharing. (11 March 2020, date last accessed).

[bib4] EC *Guidance document on the scope of application and core obligations of Regulation (EU) No 511/2014 of the European Parliament and of the Council on the compliance measures for users from the Nagoya Protocol on Access to Genetic Resources and the Fair and Equitable Sharing of Benefits Arising from their* *Utilisation* *in the Union (2016/C 313/01)* , 2016. https://eur-lex.europa.eu/legal-content/EN/TXT/PDF/?uri=CELEX:52016XC0827(01)&from=EN. (11 March 2020, date last accessed).

[bib5] EU Regulation (EU) No 511/2014 of the European Parliament and of the Council of 16 April 2014 on compliance measures for users from the Nagoya Protocol on Access to Genetic Resources and the Fair and Equitable Sharing of Benefits Arising from their Utilization in the Union, 2014.https://eur-lex.europa.eu/legal-content/EN/TXT/?uri=OJ:JOL_2014_150_R_0002. (11 March 2020, date last accessed).

[bib6] EU *Commission Implementing Regulation (EU) 2015/1866 of 13 October 2015 laying down detailed rules for the implementation of Regulation (EU) No 511/2014 of the European Parliament and of the Council as regards the register of collections, monitoring user compliance and best practices* , 2015. https://eur-lex.europa.eu/legal-content/EN/TXT/?uri=CELEX%3A32015R1866. (11 March 2020, date last accessed).

[bib7] FAO. The State of the World's Biodiversity for Food and Agriculture. Bélanger J, Pilling D (eds). Rome: FAO Commission on Genetic Resources for Food and Agriculture Assessments, 2019. http://www.fao.org/3/CA3129EN/CA3129EN.pdf (11 March 2020, date last accessed).

[bib8] Janssens D , Tindal B, Green P et al. The ECCO core Material Transfer Agreement for the supply of samples of biological material from the public collection, 2009. https://www.eccosite.org/ecco-core-mta/ (11 March 2020, date last accessed).

[bib9] McCluskey K , Barker KB, Barton HA et al. The U.S. culture collection network responding to the requirements of the Nagoya protocol on access and benefit sharing. mBio. 2017;8:e00982–17. 28811341 10.1128/mBio.00982-17 PMC5559631

[bib10] NP. Nagoya Protocol on Access to Genetic Resources and the Fair and Equitable Sharing of Benefits Arising from their Utilization to the Convention on Biological Diversity. United Nations: Secretariat of the Convention on Biological Diversity, 2011, https://www.cbd.int/abs/doc/protocol/nagoya-protocol-en.pdf.

[bib11] Overmann J , Scholz A. Microbial research under the Nagoya protocol: facts and fiction. Trends Microbiol. 2017;25:85–88. 27887771 10.1016/j.tim.2016.11.001

[bib12] Smith D , da Silva M, Jackson J et al. An explanation of the Nagoya protocol and access and benefit sharing, and its implication for microbiology. Microbiology. 2017;163:289–96. 28086069 10.1099/mic.0.000425

[bib13] Verkley G , Martin D, Smith D. MIRRI best practice manual on access and benefit sharing, 2016. https://absch.cbd.int/database/A19A20/ABSCH-A19A20-SCBD-208213. (11 March 2020, date last accessed).

