# Supplementary material for: New ECCO model documents for Material Deposit and Transfer Agreements in compliance with the Nagoya Protocol
Source: FEMS Microbiol Lett. 2020 Mar 9;367(5):fnaa044. doi: 10.1093/femsle/fnaa044 (PMC7164777; doi:10.1093/femsle/fnaa044)
Supplement: fnaa044_Supplemental_Files [file fnaa044_supplemental_files.zip › Verkley_supplementary_material_1-The_MDA_model_resubmission_accepted_changes_minor_rev.docx]

supplementary material 1 - The MDA model

**ECCO MDA model**

**Material Deposit Form and Definitions and Terms and Condition for Material Deposit Agreement (MDA)**

***for the deposit of material in the public collection***

**Part I – Deposit Form^[[1]](#footnote-1)^**

**To be filled in by the COLLECTION:**

**COLLECTION accession number:**

**Date of deposit*:**

**This could be the date the culture was received, or a later date, depending on the Collection’s procedures.*

1. **General**

**Strain identifier** (Depositor’s culture number):

**(Proposed) scientific name of the organism** (genus and/or species name):

***Is the MATERIAL a type strain?*** □ YES □ NO

**Other collection numbers** (other BRC accession numbers for this strain):

***Is the strain genetically modified?***

□ NO

□ YES →additional information is required to comply with national GMO legislation, for which the collection can provide a section with fields needed.

1. **Information on provenance of the MATERIAL, relating to the Convention on Biological Diversity** (CBD – [www.cbd.int/](http://www.cbd.int/)) **and Nagoya Protocol on Access and Benefit Sharing (ABS), and other applicable legal requirements for access to the MATERIAL**

*In order to assure compliance with all legal and regulatory requirements any information which is relevant has to be provided to and assessed and stored by the COLLECTION. Please provide as much detail as possible on the geographical origin, and terms and conditions for use of the MATERIAL as laid down in relevant documents covering the MATERIAL, including material transfer agreements (MTA), prior informed consent (PIC), mutually agreed terms (MAT) or other documents such as collecting- or export permits of landowners etc. – Be aware that if information provided is insufficient to determine whether MATERIAL is in or out of scope of ABS legislation or regulatory requirements, the BRC may have to refuse the MATERIAL.*

**B.1 Geographic origin and sampling**

**Source** **(sample)/substrate** (from which the strain was isolated):

**Country of Origin** (if applicable):

Country of origin is not applicable, because:

□ from the High seas^[[2]](#endnote-1)^ □ from region covered by the Antarctic Treaty^[[3]](#endnote-2)^ □ other reason, namely…….

**Locality**:

**GPS Coordinates**:

If the MATERIAL originates from an area beyond national jurisdiction (e.g. the High sea, area covered by the Antarctic Treaty), provide the geographic location as precisely as possible and, if collected on High sea, the name of the vessel.

**Date of sampling *in situ***:

**Sampled by** (name of the natural person and affiliation, institute, i.e. the legal person/entity on behalf of which the sample was collected):

**Is the Material in scope of ABS legislation or regulatory requirements?**

□ YES □ I do not know □ NO, because…….

**If YES, or if you obtained some form of sampling authorization (for example under local environmental protection law) or information on conditions for use of the Material, please also complete B.2**

**B.2 Documents providing evidence of legal access in the Country of Origin and providing terms for use of the MATERIAL**

***Is the original material or the source sample subject to an International Recognized Certificate of Compliance (IRCC, see definition under Part II below)?***

□ YES, the IRCC number : …………………………………

□ NO, but I am providing relevant information and/or copies of documents at my disposal, namely:

**Prior Informed Consent (PIC)** or other sampling authorization (e.g., permit):

□ YES (attach document)

□ NO, because……

□ Not applicable, because…….

Reference, document identifier(s):

**Competent authority, landowner (including i.a., nature management body) or other person who authorized sampling (e.g. PIC, or permit)**

Name and address/website:

**Mutually agreed terms (MAT) or material transfer agreements (MTA)**

Copies of documents settling details of any agreed benefit sharing arrangements or other form of agreements between initial provider and recipient (you, or person or persons before you) of the strain will be needed.

Reference, document(s) identifier(s):

1. **Isolation of the strain**

**Date of isolation**:

**Country, Locality, State**:

**Isolated by** (name of the person and affiliation, institute, i.e., the legal person/entity on behalf of which the sample was collected):

**Other relevant details of strain history**

If you did not isolate the strain, please indicate scientists and laboratories which maintained it before you: Depositor < …………<…………<……….

1. **Hazardous Properties**

**Risk Group** assignment: RG1 / RG2* / RG3* [/ RG4*]

**The strain is known to be or likely to be pathogenic to**:

Humans / Animals / Plants / other, …………..

**Biological Safety Level recommended for containment**: BSL-1 / BSL-2* / BSL-3*

*(*) as far as the collection is authorized to accept and maintain them*

*Note: Collection can add other fields here to collect scientific and technical information, e.g. on DNA sequences deposited in public repositories (DDBG/EMBL/GenBank - NCBI), particular properties, method of identification, isolation, conditions for growth and maintenance, depending on the type of organisms the collection can accept.*

1. **Conditions for use of the MATERIAL by the COLLECTION and THIRD PARTIES**

□ I refer to the information that I have provided under B and the relevant documents I am providing to the COLLECTION. I agree that the COLLECTION will supply the MATERIAL to THIRD PARTIES under conditions in agreement with that information.

□ I have not provided any information under B and the Material is not in scope of ABS requirements (B.1). I agree that the COLLECTION will supply the MATERIAL to THIRD PARTIES under the model/standard MTA of the COLLECTION and that the COLLECTION can adopt this model/standard MTA at any time.

Optional for the COLLECTION:

□ I have not provided any information under B and the Material is not in scope of ABS requirements (B.1). As the DEPOSITOR I herewith agree that the MATERIAL can be used under the following terms and conditions:

□ for any lawful purpose in research and education, including development for commercial purposes

□ for any lawful purpose in research and education, but for non-commercial purposes only

□ for any lawful purpose in research and education. Using the MATERIAL for commercial purposes will require prior consent of the Depositor

□ other, …….

**Part II – Definitions and Terms and Condition for the deposit in the public collection**

1. **Definitions**

| a. COLLECTION – acronym and address of the Collection/BRC accessioning the original MATERIAL.  b. DEPOSITOR: Natural or legal person who provides the COLLECTION with the original MATERIAL and is responsible for completion of the DEPOSIT FORM.  c. DEPOSIT FORM: Form of the COLLECTION recording essential and minimal information to fulfil the administrative, legal, technical and scientific requirements for the deposit of the MATERIAL in the public collection. The DEPOSIT FORM together with the TERMS AND CONDITIONS make up the AGREEMENT.  d. AGREEMENT: This document, which consists of the two inseparable parts: DEPOSIT FORM and TERMS AND CONDITIONS for the deposit. |  | e. COUNTRY OF ORIGIN: The country where the original MATERIAL was taken from in-situ conditions, in a natural habitat or from its original non-natural source.  f. MATERIAL: biological material originally supplied to the COLLECTION for deposit, and progeny of the original biological material.  g. COMMERCIAL PURPOSES: The use of the MATERIAL for the purpose of profit.  h. PRIOR INFORMED CONSENT (PIC) AND/OR MUTUALLY AGREED TERMS (MAT): Records generated by the Competent National Authority of a Providing Country that may be a permit or equivalent. This may also take the form of an Internationally Recognized Certificate of Compliance (IRCC) which is a type of PIC/MAT found on the ABS Clearing House. This results in a unique identifier and link and provides legal certainty about the origin of the genetic resources. (https://absch.cbd.int/). |
| --- | --- | --- |

1. **Terms and Condition**
2. DEPOSITOR declares to have acquired the MATERIAL lawfully and in compliance with applicable legislation.
3. The information that DEPOSITOR has provided in the DEPOSIT FORM is accurate, truthful, and complete and the documents relevant to ABS that DEPOSITOR is able to provide are authentic, apply to the MATERIAL, and include all relevant information on terms and conditions for use and benefit sharing, if applicable, under which DEPOSITOR acquired the MATERIAL.
4. In case after having deposited the MATERIAL the DEPOSITOR acquires new information that bears relevance to the legal status of the MATERIAL or the terms of this AGREEMENT, DEPOSITOR will provide without undue delay that information to the COLLECTION.
5. DEPOSITOR declares that the deposit of the MATERIAL does not infringe any rights of the Country of Origin or other THIRD PARTIES, including intellectual property rights, or any terms of agreements to which the MATERIAL is subject.
6. Notwithstanding rights of third parties over the MATERIAL, DEPOSITOR herewith agrees that the COLLECTION may for unlimited duration preserve, study and supply the MATERIAL to THIRD PARTIES, including other collections, under the terms and conditions specified in Part I B and/or E of the DEPOSIT FORM. DEPOSITOR understands that a fee can be charged for supply to THIRD PARTIES to help carry costs of sustaining the COLLECTION.
7. DEPOSITOR acknowledges that the COLLECTION may need to transfer the information or copied documentation to THIRD PARTIES, if required by law or ABS best practice and as far as it is not confidential. With the exception of data provided by the DEPOSITOR that are confidential, DEPOSITOR authorizes the COLLECTION to include all data and documentation in the COLLECTION’s databases and strain catalogue and DEPOSITOR also authorizes the release of the MATERIAL for distribution and associated data for public viewing

□ as soon as possible after effective deposit, or

□ after publication of the strain / or X years after effective deposit (whichever is first).

1. DEPOSITOR shall ship the MATERIAL to the COLLECTION in accordance with all applicable laws and regulations for packaging and transport. Both parties to this AGREEMENT shall follow all procedures to comply with legal requirements for handling of the MATERIAL.
2. The DEPOSITOR indemnifies the COLLECTION from any action or claim for compensation made by THIRD PARTIES and directly or indirectly related to the MATERIAL, their exploitation and/or the products obtained through them.

DEPOSITOR:

First and last name of scientist responsible for the MATERIAL:

Position:

Name Institution:

Address Institution:

First and last name of person authorized to sign on behalf:

Position:

Date and Place of signature:

Signature

**Optional additional clauses:**

In case the depositor does not need to sign the agreement:

1. By completing the DEPOSIT FORM and effectively transferring the MATERIAL in the custody of the COLLECTION the DEPOSITOR accepts the TERMS AND CONDITIONS of this AGREEMENT

In case the collection does not need to sign the agreement:

1. The COLLECTION accepts the TERMS AND CONDITIONS of this AGREEMENT by accepting the MATERIAL for deposit.

1. Often also referred to as “Accession Form”, to accession a strain is the same as to deposit it in a collection. [↑](#footnote-ref-1)
2. In the meaning of the UN Convention of the Law of the Sea: https://www.un.org/Depts/los/convention_agreements/texts/unclos/closindx.htm [↑](#endnote-ref-1)
3. The Antarctic Treaty:

   https://www.ats.aq [↑](#endnote-ref-2)
