# Supplementary material for: New ECCO model documents for Material Deposit and Transfer Agreements in compliance with the Nagoya Protocol
Source: FEMS Microbiol Lett. 2020 Mar 9;367(5):fnaa044. doi: 10.1093/femsle/fnaa044 (PMC7164777; doi:10.1093/femsle/fnaa044)
Supplement: fnaa044_Supplemental_Files [file fnaa044_supplemental_files.zip › Verkley_supplementary_material_2-The_MTA_model_resubmission_accepted_changes.docx]

supplementary material 2 - The MTA model

**ECCO MTA model**

**Scope of agreement**

This Agreement applies to the use, handling, distribution and any deposit of the MATERIAL supplied by a COLLECTION, and sent to a RECIPIENT

1. **DEFINITIONS**
2. DESCRIPTION FORM: Form recording essential and minimal information to fulfil the administrative, legal, technical and scientific requirements for the distribution of the MATERIAL. The DESCRIPTION FORM together with the TERMS AND CONDITIONS make up the AGREEMENT.
3. AGREEMENT: This document, which consists of two inseparable parts: DESCRIPTION FORM and TERMS AND CONDITIONS for the material supplied.
4. NON-COMMERCIAL: UTILIZATION of the MATERIAL for purposes including but not limited to taxonomy, basic research, education, or quality control purposes independent of the legal/commercial status of the RECIPIENT.
5. COUNTRY OF ORIGIN: the country where, according to information of COLLECTION, the original MATERIAL was taken from in-situ conditions, in a natural habitat or from its original non-natural source.
6. DEPOSITOR: Person(s) or entity that provided the COLLECTION with the original MATERIAL and as such has been added to the records of the COLLECTION, and/or listed on the Material Deposit Agreement (MDA).
7. END-USER: Scientist working with the supplied MATERIAL.
8. INTERMEDIARY: Third-party entity that makes an order on behalf of the END-USER, and to which the COLLECTION addresses the MATERIAL but that will not engage in UTILIZATION of the MATERIAL. These can be wholesalers, importers, or other type of intermediary agents, unrelated to the END-USER’s institution.
9. Prior informed consent (PIC) AND/or mutually agreed terms (MAT): Records generated by the Competent National Authority of a Providing Country that may be a permit or equivalent. This may also take the form of an Internationally Recognized Certificate of Compliance (IRCC) which is a type of PIC/MAT found on the ABS Clearing House. This results in a unique identifier and link, information on obligations and restrictions for use, and provides legal certainty about the origin of the genetic resources. (https://absch.cbd.int/).
10. LEGITIMATE EXCHANGE: The transfer of the MATERIAL between scientists working in the same Laboratory, or between partners in different Institutions collaborating on a defined joint project, for non-commercial purposes. This also includes the transfer of MATERIAL between public service culture collections and biological resource centers (BRCs) for deposit purposes. Legitimate exchange can only be conducted provided the further distribution is under MTA conditions equivalent and compatible with those in place at the supplying collection.
11. MATERIAL: Biological material originally supplied to the COLLECTION by the DEPOSITOR, and progeny of the original biological material. The MATERIAL shall not include MODIFICATIONS.
12. MODIFICATIONS: Substances produced by the RECIPIENT by using the MATERIAL, which are not the original MATERIAL, progeny, or unmodified derivatives, and which have new properties. MODIFICATIONS include, but are not limited to, recombinant DNA clones.
13. PARTY: COLLECTION and RECIPIENT (Intermediary and END-USER) are referred to together as PARTIES
14. RECIPIENT: The party to whom the COLLECTION sends the MATERIAL. In case this is not the END-USER but an INTERMEDIARY, this INTERMEDIARY has the responsibility to ensure that END-USER has appropriate and legally compliant facilities for working with the material.
15. COLLECTION: ## Insert full name, acronym and address of the Collection/BRC supplying the material.
16. uTILIZATION: To conduct research and development on the genetic and/or biochemical composition of the MATERIAL, including through the application of biotechnology.

**Terms and conditions**

.

1. **TRANSFER OF MATERIAL**

2.1 RECIPIENT agrees that all information provided to the COLLECTION or INTERMEDIARY in connection with any order for MATERIAL is accurate and complete, and complies with applicable laws and regulations.

2.2 The COLLECTION and INTERMEDIARY (where applicable) will process, package and ship the MATERIAL ordered by RECIPIENT in accordance with applicable laws and regulations to the address as stated by RECIPIENT. After receipt of the payment or confirmation of the order RECIPIENT is responsible for ensuring that all permits required for the shipment of the MATERIAL are obtained.

2.3 RECIPIENT understands that MATERIAL designated Risk Group 2 or above (as defined by the national regulations of the country where the COLLECTION is located) may cause human disease, and that MODIFICATIONS, or other MATERIAL, not so designated, may cause human disease under certain conditions. Furthermore, the RECIPIENT or INTERMEDIARY certifies that purchase of MATERIAL of Risk Group 2 or above indicates their compliance with domestic laboratory safety or other relevant laws.

2.4 RECIPIENT agrees that any handling or other activity undertaken in their laboratory with the MATERIAL will be conducted under their responsibility and in compliance with all applicable laws and regulations.

2.5 RECIPIENT therefore assures that within their laboratory (i) access to the MATERIAL will be restricted to personnel capable and qualified to safely handle said MATERIAL and (ii) RECIPIENT shall exercise the necessary care, taking into account the specific characteristics of the MATERIAL, to maintain and use it with appropriate precautions to minimize any risk of harm to persons, property, and the environment, and to safeguard it from theft or misuse.

2.6 Unless agreed in writing with the COLLECTION, RECIPIENT shall not sell, distribute or propagate for distribution, lend, or otherwise transfer the MATERIAL to any others, except a RECIPIENT involved in LEGITIMATE EXCHANGES as defined.

1. **USE OF MATERIAL**

3.1 **COLLECTION grants RECIPIENT a limited, non-exclusive right to NON-COMMERCIAL UTILIZATION of the MATERIAL in any lawful manner.** The MATERIAL is transferred for use only as specified in the accompanying terms and conditions.

3.2 For MATERIAL within scope of the Convention on Biological Diversity (CBD) or Nagoya Protocol, UTILIZATION may be limited even further and will be indicated in PIC/MAT sent by the COLLECTION accompanying the MATERIAL (See section 4.) This AGREEMENT does NOT result in a transfer of ownership of MATERIAL.

3.3 Use of the MATERIAL may be subject to third party’s rights, such as, but not limited to, intellectual property rights and the permission of the competent authority or government of the COUNTRY OF ORIGIN.

3.4 No expressed or implied rights over the MATERIAL are provided herein to the RECIPIENT, including under any patents, patent applications, trade secrets or other proprietary rights, such as a permission to use of the competent authority or government of the COUNTRY OF ORIGIN. Nothing in this AGREEMENT grants RECIPIENT any rights under any patents, propriety, intellectual property, or other rights with respect to the MATERIAL.

3.5 RECIPIENT agrees to refer to the COLLECTION, including the strain ID number(s) (unique identifier) referring to the MATERIAL as well as the COUNTRY OF ORIGIN of the MATERIAL in all publications, taking into account applicable national and international laws implementing the Nagoya Protocol to the Convention on Biological Diversity.

**4. COMPLIANCE TO NAGOYA PROTOCOL AND CBD**

4.1 The RECIPIENT shall comply with relevant domestic, regional, and international access and benefit sharing law, and for European users, especially EU Regulation 511/2014. The RECIPIENT shall use the Material in a legitimate, safe, legal, ethical and sustainable way, and in respect of the principles laid down in the CBD. Nothing in this MTA shall be construed as changing or affecting the rights and obligations of the Parties under the CBD.

4.2 The RECIPIENT agrees to abide by the PRIOR INFORMED CONSENT (PIC) and MUTUALLY AGREED TERMS (MAT) and/or IRCC and any other condition under which the MATERIAL was originally acquired and will contact the competent authority in the COUNTRY OF ORIGIN prior to any activities that might conflict with the existing PIC and MAT or any other conditions.

4.3 Any copies of documentation relevant to ABS with regard to the MATERIAL are part of the agreement, and may include a collecting permit, MAT, PIC, export permit, import permit, IRCC or other documents and will be delivered by the COLLECTION if available. However, the absence of documentation does not free RECIPIENT from due diligence obligations in terms of the seeking of documents under the EU Regulation relevant to access and benefit sharing unless the material is received from a Registered Collection^[[1]](#footnote-1)^.

**5. LIABILITY**

5.1 NO liability is to be ascribed to the COLLECTION except to the extent that damages or losses have been caused by the wilful intent or gross negligence of the COLLECTION.

5.2 COLLECTION assumes no responsibility for the loss of viability of the biological material or other adverse effects during transportation or delays. Consulting with the customs or postal authorities of the respective transit and destination countries in case of impending adverse events is the responsibility of the RECIPIENT.

5.3 Any further liability of COLLECTION, especially for damages due to improper use of the MATERIAL or for damages due to force majeure, is excluded.

**6. WARRANTY**

6.1 If RECIPIENT is not satisfied about the quality of the MATERIAL received, it will be replaced by COLLECTION free of charge, provided that RECIPIENT has filed a substantiated complaint at COLLECTION within XX days after receipt of the MATERIAL and that the claim is justified to COLLECTION’s satisfaction. Any expiration date specified on the MATERIAL shipment documentation does not constitute a warranty. The RECIPIENT may obtain a credit or full refund if COLLECTION fails to supply a viable replacement of the MATERIAL transferred.

6.2 Disclaimer of warranties. Except as expressly provided in this AGREEMENT and within the limits of the scope of the COLLECTION’s quality system, there are no representations or warranties by the COLLECTION with respect to the MATERIAL.

**7. TERMINATION**

7.1 COLLECTION may terminate this material transfer agreement concluded with RECIPIENT, if RECIPIENT breaches one or more of its obligations thereunder and fails to cure such breach within thirty (30) days after it has received a written notice by the COLLECTION specifying the breach.

7.2 Either party may unilaterally terminate this entire Agreement at any time by giving the other party written notice not less than sixty (60) calendar days prior to the desired termination date.

7.3 After termination RECIPIENT shall immediately destroy that MATERIAL, provide evidence thereof to COLLECTION and cease the use thereof.

**8. APPLICABLE LAW; JURISDUCTION**

8.1 All material transfer agreement(s) between COLLECTION and RECIPIENT and these Terms and Conditions shall be governed by and construed in accordance with the laws of the location of the COLLECTION unless otherwise indicated in the PIC/MAT (see Section 4).

8.2 In the event of a dispute arising out of a material transfer agreement, the Parties shall seek to resolve such dispute amicably. In case the Parties fail to settle the dispute amicably within four (4) weeks after the dispute has been raised by one Party to the other Party in writing, the dispute may be brought in the competent courts of the relevant Country, who shall have exclusive jurisdiction.

1. http://ec.europa.eu/environment/nature/biodiversity/international/abs/pdf/Register%20of%20Collections.pdf [↑](#footnote-ref-1)
