# Supplementary material for: New ECCO model documents for Material Deposit and Transfer Agreements in compliance with the Nagoya Protocol
Source: FEMS Microbiol Lett. 2020 Mar 9;367(5):fnaa044. doi: 10.1093/femsle/fnaa044 (PMC7164777; doi:10.1093/femsle/fnaa044)
Supplement: fnaa044_Supplemental_Files [file fnaa044_supplemental_files.zip › Verkley_supplementary_material_3-The_MTA_model_Annex_1_resubmission_accept_change.docx]

supplementary material 3 - The MTA model Annex 1

**Annex 1: clauses FOR COMMERCIAL USE**

Note: The use clauses above are, by default, for non-commercial use of the material. For some material in the collections permission for commercial use can only be granted by the depositor or the country of origin.

For other material this is not the case and the collection itself could grant permission for commercial utilization, and the following changes can be made to build an MTA fit for that purpose.

1. This definition can be added to the definition section and replace the non-commercial use definition:

COMMERCIAL: UTILIZATION of the MATERIAL for the purpose of profit generation.

1. This phrase could be used to replace 3.1:

**COLLECTION grants RECIPIENT a limited, non-exclusive right to COMMERCIAL UTILIZATION of the MATERIAL in any lawful manner.** The MATERIAL is transferred for USE only as specified in the accompanying terms and conditions.

Additional clause:

RECIPIENT will notify the COLLECTION at least 12## weeks before filing a patent application or a similar intellectual property right involving the MATERIAL or its use and will enter into a separate commercialization agreement either with the COUNTRY OF ORIGIN, DEPOSITOR, or COLLECTION depending on the origin of the strain.
