# Supplementary material for: New ECCO model documents for Material Deposit and Transfer Agreements in compliance with the Nagoya Protocol
Source: FEMS Microbiol Lett. 2020 Mar 9;367(5):fnaa044. doi: 10.1093/femsle/fnaa044 (PMC7164777; doi:10.1093/femsle/fnaa044)
Supplement: fnaa044_Supplemental_Files [file fnaa044_supplemental_files.zip › Verkley_supplementary_material_4-The_MTA_model_Annex_2_resubmission_accepted_changes.docx]

supplementary material 4 - The MTA model Annex 2

**Annex 2: Description FORM**

| Supplied material | | | | | | Documents | | | |
| --- | --- | --- | --- | --- | --- | --- | --- | --- | --- |
| Strain Identifi-cation number | Organism type | Organism species | Biological Safety Level (BSL) | Geographic origin | Nagoya compliance: condition* | PIC | MAT | IRCC | Other** |
|  |  |  |  |  |  |  |  |  |  |
|  |  |  |  |  |  |  |  |  |  |
|  |  |  |  |  |  |  |  |  |  |
|  |  |  |  |  |  |  |  |  |  |
|  |  |  |  |  |  |  |  |  |  |
|  |  |  |  |  |  |  |  |  |  |
|  |  |  |  |  |  |  |  |  |  |
|  |  |  |  |  |  |  |  |  |  |

* Condition of strain collection:

1) - prior to Dec. 29, 1993^[[1]](#footnote-1)^

- in High seas or region covered by the Antarctic Treaty

- in Country that is non-party to the CBD

- in Country that is a party to CBD and to NP, but provides free access to their genetic resources.

No documents required

2) - in Country that is a party to CBD, but not NP

- in Country that is party to CBD and to NP and does not provide free access but the sample was collected before October 12, 2014 or before the national ABS legislation went into force in that Country

Documents recommended, in any form which confirms that the strain was legally acquired (these may be permits or written permission statements from a body entitled)

3) - in Country that is a party to CBD and a party of NP and does not provide free access and sample was collected after the national ABS legislation went in force in this country

Document required that are issued in whatever valid form under national ABS legislation in the country of origin, typically this could be one or more of the following: PIC – Prior Informed Consent; MAT – Mutually Agreed Terms; MTA – Material Transfer Agreement; IRCC - Internationally Recognised Certificate of Compliance

** MTA, or other documents such as collection permits from National parks, landowners etc., not based on ABS legislation

1. in as far as the country of origin did not issue permits based on sovereign rights exercised from the UN resolution on Permanent Sovereignty over Natural Resources of Dec. 14, 1962 [↑](#footnote-ref-1)
